# Notes and News

**Published:** 1890-04-19

**Authors:** 


### Notes and News.

Collected and Communicated.

On Wednesday, March 26th, the children of St. Paul's Sunday School, Wokingham, sent a cuckoo clock, the result of two offertories, to the children in Reading Hospital; it bears the following inscription: "Presented to the Children's Ward, Royal Berks Hospital, by the children of St. Paul's Sunday School, Wokingham, Christmas, 1889." This is the second gift received at the hospital through the kind efforts of the Rev. F. Leslie.

On the 15th Mr. Gerald Loder opened a bazaar at Hove Town Hall, in aid of the Brighton and Hove Dispensary. This institution has lately received three donations of £2,500 each. Mrs. Carr Burton gave the first two donations, and lately Mrs. Macdonald has given a similar sum, on condition that her name is placed above two of the beds in the wards. This generosity shows that the inhabitants of Hove have faith in the good done by the dispensary.

The annual report of the Hertford Infirmary states that in the year 1889 there were no fewer than 2,259 patients under treatment either in or out of the house. Of these the large number of 1,019 were cured, and 479 relieved; so that the magnitude of the work it at once apparent. During the year the collections from places of worship in the sphere of operations embraced in the work, only reached the sum of £254 8s. 11d.; the donations amounted to £208 12s. 4d., and in all the collecting boxes only five reached the sum of £1 and upwards. The expenditure exceeds the income for last year by nearly £300.

A correspondent of the *Macclesfield Courier* calls attention to reported mismanagement at the infirmary, which he hints arises from "petticoat" influence, which prevents the house surgeon occupying a proper position. But if this correspondent had taken the trouble to analyse the expenditure of the institution according to facts given in the *Hospital Annual,* he would have seen that the housekeeping is less than that in similar institutions, but the medical and surgical expenditure is greater. That the house surgeon should have resigned after only six months' service is to be regretted, and certainly the constant changes of the staff point to a fault somewhere, and the governors should bestir themselves to find where the fault lies.

The 17th National Conference of Charities and Correction, will be held at Baltimore, Maryland, from May 14th to 21st. The list of papers to be read, and subjects to be discussed, is of great interest, and includes Hospitals, Training Schools for Nurses, Charity Organization, and the Care of the Insane. Delegates and specialists from England will be present, and both a pleasant and an instructive conference is expected. The objects of local interest in Baltimore are sufficient to make a visit to that city attractive, for do they not include the John Hopkins University and Hospital?

The annual report of the Whitehaven and West Cumberland Infirmary states, that last year the number of in-door patients was 229, being exactly the same as the previous year, and the average stay of each was 46 days, or 3⅔ days longer than the average for 1888. The outside patients numbered 1,631 of which about 400 were visited at their own homes. The income from ordinary sources has again been sufficient to meet all the demands made upon the funds, and to carry forward a small balance, the total receipts being £1,427 17s. 7d., and the expenditure £1,349 13s. 10½d., the former being £126, and the latter £60 in excess for 1888.

A very excellent but quiet little hospital is the North-Eastern Hospital for Children, which has a number of Quakers on its committee, and perhaps for this reason does not push itself before the public and take the front place it deserves. A correspondent writes to us: "It is a very pretty, bright hospital. I saw two wards with 27 beds in each, and they have about one thousand out-patients a-week. One cot is supported by the Sunday School children of Shoreditch Church. I received such a pleasant welcome from Miss Curno, the matron, and found so much that was interessing to see, that I recommend all those who do not know this institution to visit it at once."

Alderman W. D. Stephens, has revived interest in the scheme of a new infirmary for Newcastle. At the House Committee on April 3rd he proposed that the Moor Lodge site for a cancer hospital should be secured as a site for a new infirmary, and that on the ground where the infirmary now stands a Town Hall should be erected. It is to be remarked that though Mr. Stephens put part of his proposal—that relating to the new infirmary—in the form of a resolution, he did so purely in a tentative way, and very readily withdrew his motion on finding that it was regarded as somewhat premature. Reference was made to the bequest of £10,000 by the late Mr. Fleming, which would form a nucleus for carrying out this scheme.

The annual report of the Medical Mission to the Dera Ghazi Khan in Central India is a foreign voice indeed. The report very wisely commences with a map of the district, for we are sure few people in England know much of the Biloches, though, thanks to Rudyard Kipling, many of us have been rubbing up our Indian geography lately. The report tells of increased work: 8,375 out-patients and 163 in-patients, with 91 major operations, 419 minor operations, and only 5 deaths. There is a Zenana Dispensary, under Mrs. Ghulam Ladir Shah, which is open three days a-week. Mr. Jukes, who is at the head of the mission, lately visited some of the neighbouring districts. He writes: "We followed the dry bed of the Sori torrent for several miles to visit some sulphur springs at a place called Yinda Pir (the living saint). A Faqir has lived here for seven generations, and as no grave appears, the presumption to these untutored people is, that the predecessors of the present occupier are still living. Many forms of sickness are said to be cured by these springs, and leprosy is said to be amongst the number. We afterwards came to several other places where the waters are decidedly sulphurous and milky in appearance, some quite hot."

The following vacancies are announced this week, the amount of the salary in each case being given after the name of the institution:—

**Resident Medical Officers.**

Birmingham Queen's Hospital, House Physician—£50, board, etc.
Birmingham Queen's Hospital, House Surgeon—£50, board, etc.
Colney Hatch Asylum, Medical Superintendent—£1,000, house, etc.
York County Hospital, Senior House Surgeon—£100, board, etc.
Bolton Infirmary, Junior House Surgeon—£100, board, etc.
Norfolk Hospital, Assistant House Surgeon—Board, etc.
Chorlton-upon-Medlock Dispensary, House Surgeon—£100, board, etc.
Lancaster County Asylum, Pathologist—£700, board, etc.
North-West London Hospital, Medical Officer—£50, board, etc.
North-West London Hospital, Assistant Medical Officer—Board, etc.
St. Saviour's Union, Assistant Medical Officer—£100, board, etc.

**Non-Resident Medical Officers.**

Walsall Friendly Societies, Medical Officer—£200.
Hendon Sanitary Authority, Sanitary Medical Officer—£100.
Glasgow Western Medical School, Lecturer on Surgery.
St. Marylebone Dispensary, Honorary Physician.
Clerkenwell Vestry, Analyst—£100.

Miss Clara Waddington, last surviving sister of the late Dean Waddington, of Durham, has, by her will, bequeathed the handsome sum of £2,000 to the funds of the Durham County Hospital. Dean Waddington himself was a great benefactor of the institution, one of the wards being named "Waddington Ward."

The festival dinner of King's College Hospital, takes place on the 24th, the Archbishop of Canterbury in the chair. Last year a special effort was made to raise funds, and one of the closed wards was re-opened; it is hoped this year that the other ward, which is yet closed, may also be opened. The hospital entered the current year free of debt, and if only the dinner be well supported by generous friends, it hopes to commence next year in a similar happy condition.

Dr. Julius Nelson, of New York, has examined four thousand of his dreams, and finds that evening and nocturnal dreams are connected with events of the day; but the latter have more of the terrifying element. The pleasantest and most remarkable dreams are those of the morning, after the rest of the brain. We can echo the experiences of the American physician, but surely it is only a New York doctor who could be so practical as to analyse dreams! There is a remarkable fascination about the subject, but the more the attempt is made to talk about or to study the subject, the farther away and the fewer become the dreams. Unless Dr. Nelson stops his sacrilegious examination, this last "trailing glory" will vanish away with the fairies, and closer and closer about us will settle the stifling atmosphere of this work-a-day world.

The quarterly report of the Devonshire Hospital and Buxton Bath Charity states that during the three months ending March 31st, 1890, two hundred and eighty-three in-patients have been admitted to the hospital. Of this important number one hundred and seventy-six were discharged "improved"; four as "no better"; one "by own request"; one as found to be "infested with vermin"; one was sent away for "drunkenness"; six had died, and 94 remained on the books at the end of the quarter. Of the six deaths only one could be classed as strictly a patient of the hospital under its rules and regulations, the death being due to pulmonary disease, more or less connected with the wearing effects of rheumatism. The other five deaths were due: the first to pyæmia from blood poisoning, and general exhaustion; the second, to pneumonia, sent from home as having been convalescent; the remaining deaths having been from severe accidents, received as such into the hospital. The death of the Hon. and Rev. F. R. Grey, who was a vice-president of the hospital is referred to with regret.

The North Riding Infirmary at Middlesbrough continues to flourish. On account of the increasing demand for in-patient accommodation, and at the urgent request of the medical staff, the ward closed in 1884 through failing revenue has been refurnished, at a cost of £160 14s. 3d., and re-opened for the admission of patients. It is calculated that the annual expenditure will thereby be increased by at least £200. The statement of accounts for the year ending December 31st, 1889, show the institution to be in a most satisfactory financial position, having a balance of £1,263. The secretary (Mr. Angus Macpherson) states that in addition to the handsome donation of Sir Raylton Dixon, that gentleman had intimated that he intended founding a child's cot in commemoration of the Princess Alexandra's visit to Middlesbrough, and he intended obtaining the consent of the Princess to call it the Alexandra cot.

Mr. Gladstone is usually the most correct of men, but some correspondents have doubted his statement that the position of the physician is so much better now-a-days than formerly. In the earliest times the doctor was a magician, and as such commanded unbounded fear and respect; but coming to more modern days, Lady Verney points out that Dr. Mayerne, of Charles I. time, is spoken of with great respect in contemporary letters, and his daughter was considered in court circles the best match in London. Mr. Besant has drawn an unpleasant picture of the sycophantish family physician of the eighteenth century, but let us hope the picture was representative of a very small class of men. One of the weekly papers has been giving types of medical men, which are poor reading and contain nothing new. Sarcasms which date from Swift have lost their sting by this time because they are worn threadbare and no longer applicable.

So the unfortunate creature known as the elephant man has been at last freed from the burden of the diseased flesh which had truly been an awful earthly load to him. Probably now we shall know the real nature of the disease which so disfigured him, and we have yet to learn the cause of his death. It will be remembered that some four years ago the poor creature was being shown about the country when his case attracted the attention of the authorities of the London Hospital, and they offered him a private room for life within their walls. The man was very grateful, for he felt his disfigurement keenly. At first he kept himself to himself utterly at the London, but, gradually finding the loneliness wearisome, he welcomed visitors to his little room, especially if they brought him tobacco. The Prince and Princess of Wales on the occasion of their last visit to The London went to see the elephant man, but wisely left their daughters in the children's ward the while.

Perhaps the present inclination to popularize medicine was never before so strongly shown as in the present series of articles in the *Times* on influenza. Column after column of the history of the epidemic, of the symptoms and their treatment, has appeared during the last week, and the medical side of the question was thoroughly gone into in a popular manner. The strongest advocates of breaking down all foolish reserve and etiquette have felt a little aghast at the outspoken treatment of the subject, and this may be exemplified by the fact that we do not choose to quote here the paragraph on hœmorrhage; its only fit place would be in the "Practitioner's Outlook." The definition of the disease is good: "Influenza is a specific fever, epidemic and often pandemic, of sudden onset and short duration, attended with loss of appetite and very great prostration, associated often with more or less severe catarrh, neuralgic pains, or gastro-intestinal disturbance, and especially liable to be complicated by severe respiratory affections, to which the mortality of the disease is chiefly due."

